# Association between history of cannabis use and outcomes after total hip or knee arthroplasty: a systematic review and meta-analysis

**DOI:** 10.3389/fpubh.2024.1377688

**Published:** 2024-05-17

**Authors:** Guangyao Yang, Feng Li, Qiuyuan Wang, Youwen Liu, Jiayi Guo, Chen Yue

**Affiliations:** Luoyang Orthopedic Traumatological Hospital, Luoyang, China

**Keywords:** Cannabis, total hip arthroplasty, total knee arthroplasty, postoperative outcomes, drug abuse and addiction

## Abstract

**Background:**

Cannabis use may be increasing as countries legalize it and it becomes socially acceptable. A history of cannabis use may increase risk of complications after various kinds of surgery and compromise functional recovery. Here we systematically reviewed and meta-analyzed available evidence on how history of cannabis use affects recovery after hip or knee arthroplasty (THA/TKA).

**Methods:**

The PubMed, EMBASE, and Web of Science databases were comprehensively searched and studies were selected and analyzed in accordance with the PRISMA guidelines. The methodological quality of included studies was assessed based on the Newcastle-Ottawa Scale, while quality of evidence was evaluated according to the “Grading of recommendations assessment, development, and evaluation” system. Data on various outcomes were pooled when appropriate and meta-analyzed.

**Results:**

The systematic review included 16 cohort studies involving 5.91 million patients. Meta-analysis linked history of cannabis use to higher risk of the following outcomes: revision (RR 1.68, 95% CI 1.31–2.16), mechanical loosening (RR 1.77, 95% CI 1.52–2.07), periprosthetic fracture (RR 1.85, 95% CI 1.38–2.48), dislocation (RR 2.10, 95% CI 1.18–3.73), cardiovascular events (RR 2.49, 95% CI 1.22–5.08), cerebrovascular events (RR 3.15, 95% CI 2.54–3.91), pneumonia (RR 3.97, 95% CI 3.49–4.51), respiratory failure (RR 4.10, 95% CI 3.38–4.97), urinary tract infection (RR 2.46, 95% CI 1.84–3.28), acute kidney injury (RR 3.25, 95% CI 2.94–3.60), venous thromboembolism (RR 1.48, 95% CI 1.34–1.63), and deep vein thrombosis (RR 1.42, 95% CI 1.19–1.70). In addition, cannabis use was associated with significantly greater risk of postoperative transfusion (RR 2.23, 95% CI 1.83–2.71) as well as higher hospitalization costs.

**Conclusion:**

History of cannabis use significantly increases the risk of numerous complications and transfusion after THA or TKA, leading to greater healthcare costs. Clinicians should consider these factors when treating cannabis users, and pre-surgical protocols should give special consideration to patients with history of cannbis use.

## Introduction

Cannabis, extracted from plants of the *Cannabis* genus, contains cannabinoids, which have been used in the traditional medicine of various countries against pain, inflammation, spasms, depression and asthma (1, 2). At the same time, cannabinoids can have wide-ranging effects on cognitive, cardiovascular, respiratory, nervous, and psychological functions, and they can induce addiction (3). As a growing number of countries legalize cannabis use, whether for recreation or specific medical purposes, concerns are growing about how history of cannabis use affects how people respond to medical treatments. For example, some studies have suggested that individuals with cannabis use disorder are at higher risk of various complications than other individuals after diverse elective surgeries (4, 5).

THA and TKA are the most successful surgeries for patients with severe painful, deformed, and damaged joints. In the United States, the annual demand for hip arthroplasty is expected to reach 710,000 and demand for knee arthroplasty to reach 1.2 million by 2040 (6). Several studies have linked history of cannabis use to increased risks of various postoperative complications such as cardiovascular events (7–9), pneumonia (7, 9), venous thromboembolism (7, 9, 10), and acute kidney injury following THA/TKA. Total joint arthroplasty patients with history of cannabis use may be also at higher risks of postoperative transfusion (7, 9) and morphine consumption (11–16). However, a comprehensive understanding of the associations between history of cannabis use with complications and poor outcomes after hip or knee arthroplasty is still lacking.

We are not aware of any published systematic review or meta-analysis in this field. Therefore, we systematically reviewed the available evidence on how history of cannabis use affects the following postoperative outcomes after THA/TKA: systemic complications, prosthetic complications, transfusion, morphine consumption, pain, and hospitalization costs. Our findings may help clinicians and patients predict prognosis after joint arthroplasty surgery, and they may guide future research on the effects of cannabis use on surgical outcomes more generally.

## Method

This systematic review and meta-analysis, whose protocol was registered on PROSPERO under accession number CRD42023472424, were performed and reported in accordance with the “Preferred Reporting Items for Systematic Reviews and Meta-Analyses” (PRISMA) (17) and “Assessing the methodological quality of systematic reviews” (AMSTAR) (18) guidelines. Ethics approval was not sought because we retrospectively analyzed studies previously published in peer-reviewed journals.

### Search strategy

Two researchers independently searched the databases of PUBMED, Embase, and Web of Science in August 2023 using combinations of MESH terms and keywords. The PUBMED search strategy were as follows: (“cannabis”[MeSH Terms] OR “cannabis”[All Fields] OR “marijuana”[All Fields]) AND (“arthroplasty, replacement”[MeSH Terms] OR “hip arthroplasty”[All Fields] OR “hip replacement”[All Fields] OR “knee arthroplasty”[All Fields] OR “knee replacement”[All Fields]).

### Study selection

To be eligible for inclusion, studies had to report original research comparing outcomes after THA/TKA between patients with or without a history of cannabis use, and the outcomes had to contain at least one of the following: systemic complications, prosthetic complications, transfusion, morphine consumption, pain, and hospitalization costs. Studies were excluded from the review if the control group involved any additional interventions, involved surgeries other than hip or knee arthroplasty, or did not report relevant outcomes. No restrictions were imposed on study design, language or publication date. References lists in potentially eligible studies were also searched manually in order to identify additional studies.

Potentially relevant studies were imported into Zotero 5.0,^1^ duplicate publications were removed, and two researchers independently screened studies based on titles and abstracts. They then reviewed the full texts of the remaining articles in order to decide on the final set. Discrepancies were resolved through discussion with a third author.

### Data extraction

Two researchers independently extracted the following data from included studies: author names, publication year, study design, sample size, numbers of patients undergoing THA/TKA, how cannabis use was defined. Outcomes of interest included prosthetic complications, systemic complications, transfusions, postoperative morphine consumption, postoperative pain, and hospitalization costs. Discrepancies were resolved through discussion with a third author.

### Assessment of study quality and evidence quality

The methodological quality of included studies was assessed using the Newcastle-Ottawa Scale (19), which is a validated, widely used tool for evaluating the quality of observational studies with a total point of nine. The scoring items included (1) the methods used to select the study groups (0–4 points), (2) the comparability of cases and controls (0–2 points), and (3) the method used to ascertain the outcome of interest (0–3 points).

The overall quality of evidence for each outcome was categorized as *high, moderate, low,* or *very low* according to the “Grading of recommendations assessment, development, and evaluation” system (20).

Assessments were made independently by two researchers, and discrepancies were resolved through discussion with a third researcher.

### Statistical analysis

Outcomes were synthesized qualitatively if data could not be directly compared across studies. Outcomes for comparable data were pooled between studies and meta-analyzed using a random-effects model in RevMan 5 software (The Cochrane Collaboration, Oxford, United Kingdom). Pooled results were reported as relative risks (RRs) for dichotomous outcomes or as weighted mean differences (WMDs) for continuous outcomes. In both cases, accompanying 95% confidence intervals (CIs) were also calculated. Heterogeneity across studies was assessed using the I^2^ test, with I^2^ > 50% defined as substantial heterogeneity (21).

## Results

**Figure 1:**
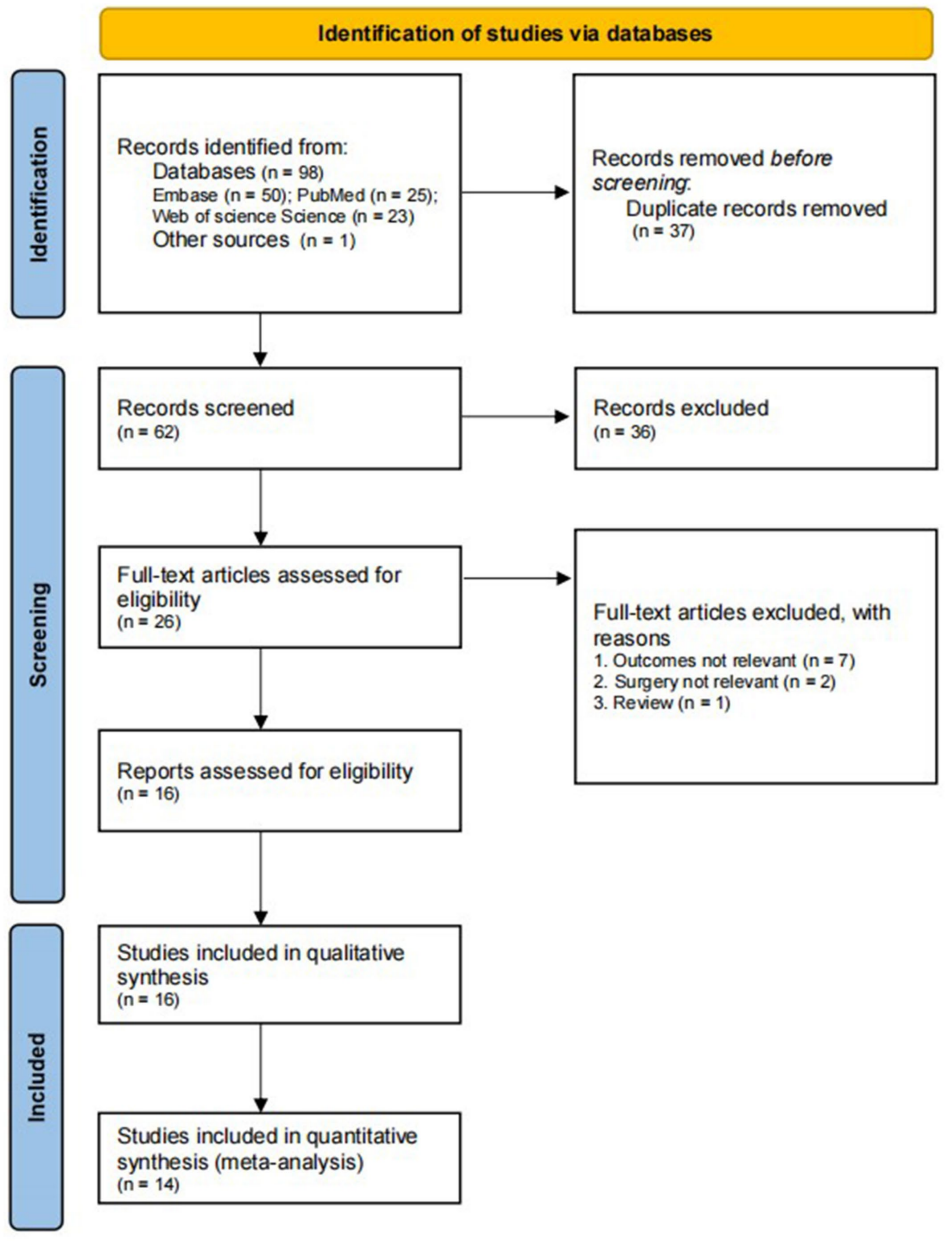
Flowchart of study selection.

Of 99 potentially relevant publications, 37 were first excluded because they were duplicates. Next, another 36 were excluded because they did not fulfill the inclusion criteria based on titles and abstracts (Figure 1). The remaining 26 publications were read in full, leading to the inclusion of 16 in the final review. Two of these were prospective (13, 22), while the others were retrospective (7–12, 14–16, 23–27). All studies were published in 2018 or later, and 10 were published in 2021 or later (7–9, 13–16, 25–27). These studies analyzed 42,602 individuals with history of cannabis use and 5,865,258 individuals who reported no cannabis use as a control group. Six studies analyzed patients who underwent either THA/TKA (11, 13, 14, 22, 24, 27), while five each examined patients after only one or the other type of surgery. Nine studies defined the history of cannabis use as “cannabis use” (12–16, 23–25, 27), five as “cannabis use disorder” (7–10, 26), one as “tetrahydrocannabinol use” (22) and one as “cannabinoid use” (11). The methodological quality of these studies ranged from 6 to 9 on the Newcastle-Ottawa Scale.

The detailed description of the study characteristics is shown in Table 1, and summary of outcomes data is shown in Table 2.

**Table 1:** Characteristics of the included studies.

Study	Country	Design	No. cannabis users, controls	Surgery	Def. cannabis use	NOS
Law et al. (23)	USA	RC	18,875/2,718,023	TKA	Cannabis use	6
Hickernell et al. (11)	USA	RC	81/162	TKA/THA	Cannabinoids use	6
Vakharia et al. (10)	USA	RC	3,680/14,708	TKA	Diagnosis with cannabis use disorder	7
Jennings et al. (12)	USA	RC	71/71	TKA	Cannabis use	6
Runner et al. (22)	USA	PC	32/163	TKA/THA	Tetrahydrocannabinol	6
Aleissa et al. (24)	USA	RC	49/100	TKA/THA	Cannabis use	6
Vakharia et al. (9)	USA	RC	3,842/19,188	THA	Diagnosis with cannabis use disorder	7
Zalikha et al. (8)	USA	RC	5,390/ 2,833,351	THA	Diagnosis with cannabis use disorder	7
Kirchner et al. (25)	USA	RC	534/534	THA	Cannabis use	7
Sambandam et al. (26)	USA	RC	278/231,375	TKA	Diagnosis with cannabis use disorder	8
Weisberg et al. (7)	USA	RC	9,260/46,293	TKA	Diagnosis with cannabis use disorder	9
Sharma et al. (27)	USA	RC	24/24	TKA/THA	Cannabis use	7
Ong et al. (15)	USA	RC	156/936	THA	Cannabis use	7
Jennings et al. (13)	USA	PC	46/46	TKA/THA	Cannabis use	6
Hegde et al. (14)	USA	RC	210/210	TKA/THA	Cannabis use	7
Hegde et al. (16)	USA	RC	74/74	THA	Cannabis use	7

NOS, score on the Newcastle-Ottawa Scale; NR, not reported; PC, prospective cohort; RC, retrospective cohort; THA, total hip arthroplasty; TKA, total knee arthroplasty, Def., definition.

**Table 2:** Summary of outcomes of the included studies.

Study	Postoperative complications		Postoperative morphine consumption, OE	Postoperative transfusion	Postoperative pain	Hospitalization costs, USD
	Prosthetic	Systemic				
Law et al. (23)	Mechanical loosening 1.4%/0.9%; Periprosthetic fracture 0.2%/0.1%; Periprosthetic joint infection 2.9%/1.4%	NR	NR	NR	NR	NR
Hickernell et al. (11)	NR	NR	121.7 ± 76.3/131.5 ± 111.7	NR	3.0 ± 2.6/2.5 ± 2.8	NR
Vakharia et al. (10)	NR	Venous thromboembolism 2.8%/1.8%; Deep vein thrombosis 2.4%/ 1.5%; Pulmonary embolism 1.0%/0.6%	NR	NR	NR	19,155.45/16,315.00
Jennings et al. (12)	NR	NR	137 ± 104/146 ± 117	NR	NR	NR
Runner et al. (22)	NR	NR	NR	NR	THA (3.45/3.50); TKA (3.40/3.79)	NR
Aleissa et al. (24)	NR	NR	NR	NR	4.8 ± 1.9/ 3.6 ± 2.3	NR
Vakharia et al. (9)	Dislocation 4.9%/1.8%; Mechanical loosening 1.8%/0.9%; Periprosthetic fracture 1.4%/0.7%; Periprosthetic joint infection 2.1%/0.9%; Revision 5.0%/2.7%	Cerebrovascular accidents 1.1%/0.3%; Pneumonia 3.9%/1.1%; Respiratory failure 1.3%/0.3%; Myocardial infarction 0.6%/0.2%; Acute kidney injury 4.0%/1.2%; Urinary tract infection 4.3%/2.2%; Ileus 0.4%/0.2%; Deep vein thrombosis 1.7%/1.1%; Pulmonary embolism 0.6%/0.5%; Venous thromboembolism 2.1%/1.4%	NR	3.1%/1.3%	NR	16,938.00/16,023.00
Zalikha et al. (8)	NR	Cardiac complication 0.93%/0.67%; Genitourinary complication 1.95%/0.56%; Deep vein thrombosis 0.19%/ 0.20%; Pulmonary embolism 0.03% /0.19%	NR	NR	NR	59,570/53,316
Kirchner et al. (25)	NR	NR	NR	NR	NR	17,847 ± 10,024/16,284 ± 7,025
Sambandam et al. (26)	Revision 0.50%/2.3%	NR	NR	NR	NR	NR
Weisberg et al. (7)	Periprosthetic fractures 0.63%/0.29%; Mechanical loosening 2.07%/1.14%; Periprosthetic joint infection 5.22%/2.66%; Dislocation 0.98%/0.63%	Pneumonia 4.11%/0.99%; Respiratory failures 1.49% /0.35%; Myocardial infarctions 0.73%/0.19%; Ileus 0.69%/ 0.24%; Cerebrovascular accidents 1.00%/0.32%; Acute kidney injuries 4.78%/1.47%; Urinary tract infections 6.50%/2.96%; Venous thromboemboli 3.32%/2.28%; Deep vein thromboses 2.19%/1.69% Pulmonary emboli 1.05%/0.92%	NR	2.20%/1.08%	NR	24,292.15/20,616.40
Sharma et al. (27)	NR	NR	NR	NR	4.7 ± 2.11/4.9 ± 2.34	NR
Ong et al. (15)	NR	NR	137.8 ± 132.0/123.9 ± 111.4	NR	NR	NR
Jennings et al. (13)	NR	NR	78.7 ± 58.5/70.4 ± 46.3	NR	4.1 ± 1.9/3.4 ± 1.6	NR
Hegde et al. (14)	NR	NR	109.0 ± 5.5/99.7 ± 5.4	NR	NR	NR
Hegde et al. (16)	NR	NR	102.4 ± 13/101 ± 9.3	NR	NR	NR

OE, opioid equivalents; NR, no report. Values are mean ± SD or risk ratio.

### Prosthetic complications

**Figure 2:**
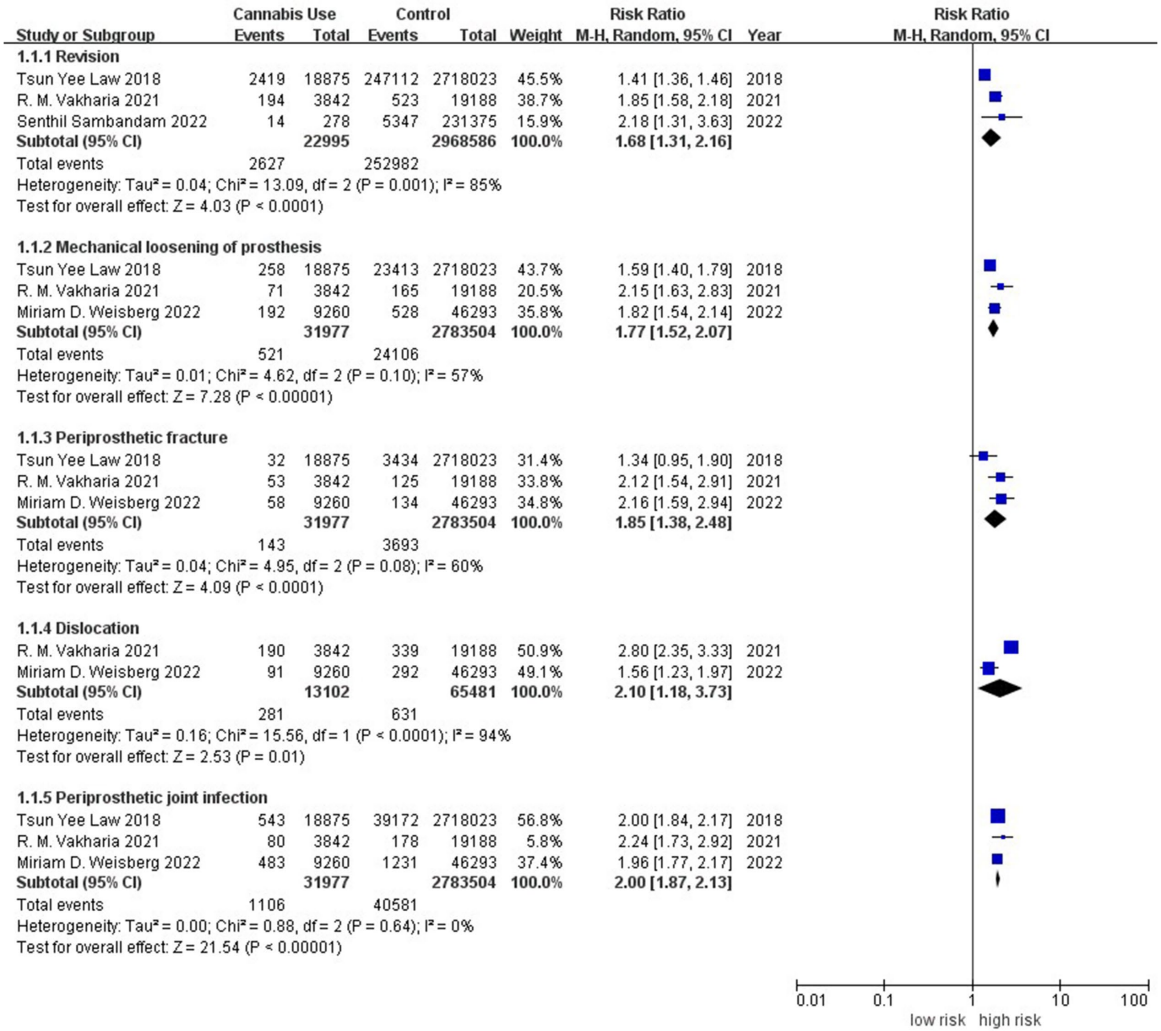
Forest plot of the risk of prosthetic complications between patients with or without a history of cannabis use after total knee or hip arthroplasty. CI, confidence interval; M–H, Mantel–Haenszel; Random, random-effects model.

Three studies involving 2,991,581 individuals investigated the rate of revision (9, 23, 26). Meta-analysis associated history of cannabis use with significantly higher revision rate (RR 1.68, 95% CI 1.31–2.16; I^2^ = 85%; Figure 2). Similar results were obtained for incidence of mechanical loosening of prosthetic (RR 1.77, 95% CI 1.52–2.07; I^2^ = 57%) (7, 9, 23), incidence of periprosthetic fracture (7, 9, 23) (RR 1.85, 95% CI 1.38–2.48; I^2^ = 60%) and incidence of periprosthetic infection (RR 2.00, 95% CI 1.87–2.13; I^2^ = 0%), based on three studies involving 2,815,481 individuals (7, 9, 23).

Meta-analysis of two studies (7, 9) involving 78,583 individuals linked history of cannabis use to significantly higher dislocation rate (RR 2.10, 95% CI 1.18–3.73; I^2^ = 94%).The evidence level was judged to be low for all these meta-analyses (Appendix 1).

### Systemic complications

**Figure 3:**
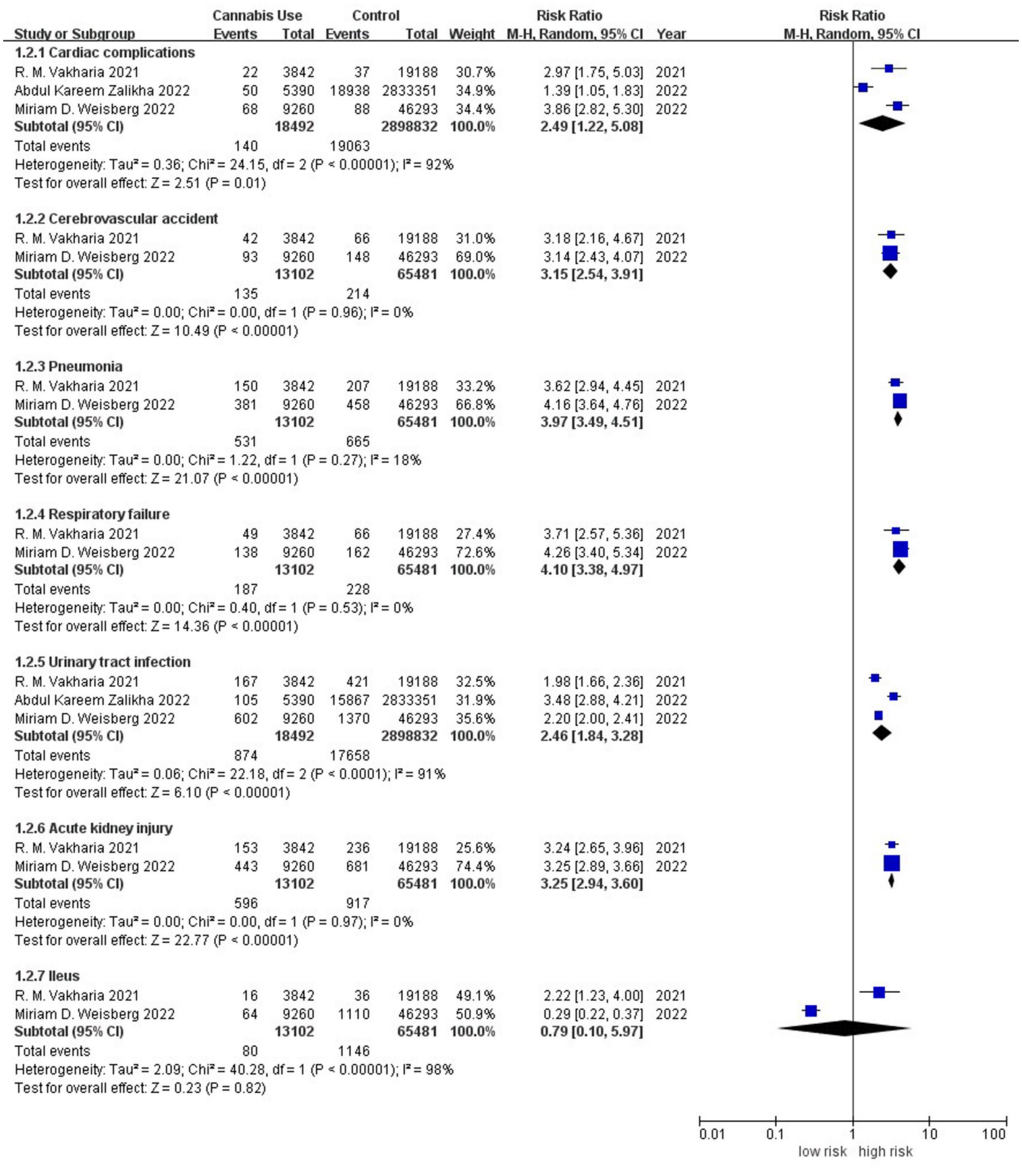
Forest plot of the risk of system complications between patients with or without a history of cannabis use after total knee or hip arthroplasty. CI, confidence interval; M–H, Mantel–Haenszel; Random, random-effects model.

Meta-analysis of three studies involving 2,917,324 individuals associated history of cannabis use with significantly higher incidence of cardiovascular complications (RR 2.49, 95% CI 1.22–5.08; I^2^ = 93%; Figure 3), for which the evidence level was low (Appendix 1). Meta-analysis of two studies (7, 9) involving 78,583 individuals linked cannabis use to significantly higher incidence of cerebrovascular complications (RR 3.15, 95% CI 2.54–3.91; I^2^ = 0%), for which the evidence level was moderate (Appendix 1).

Meta-analysis of two studies (7, 9) involving 78,583 individuals associated history of cannabis use with significantly higher incidence of postoperative pneumonia (RR 3.97, 95% CI 3.49–4.51; I^2^ = 18%) and postoperative respiratory failure (RR 4.10, 95% CI 3.38–4.97; I^2^ = 0%); the evidence level in both cases was moderate (Appendix 1). Meta-analysis of the same two studies (7, 9) linked cannabis use to significantly higher incidence of acute kidney injury (RR 3.25, 95% CI 2.94–3.60; I^2^ = 0%), for which the evidence level was moderate (Appendix 1); but it suggested no difference in incidence of postoperative ileus between patients (7, 9) with or without history of cannabis use (RR 0.79, 95% CI 0.10–5.97; I^2^ = 98%), though the evidence level for this outcome was very low (Appendix 1).

Meta-analysis of three studies (7–9) involving 2,917,324 individuals linked history of cannabis use to significantly higher incidence of urinary tract infection (RR 2.46, 95% CI 1.84–3.28; I^2^ = 93%), though the evidence level for this outcome was low (Appendix 1).

**Figure 4:**
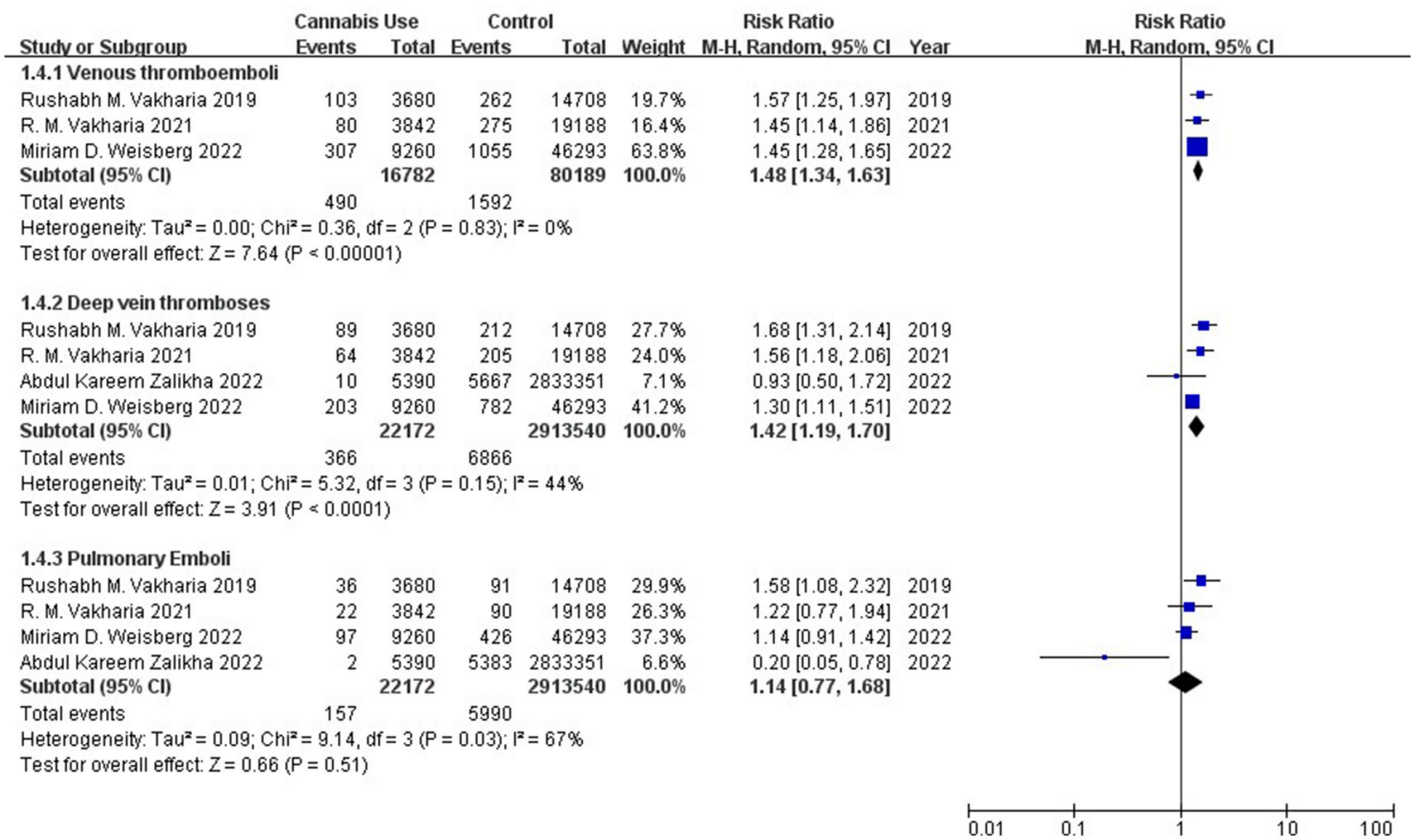
Forest plot of the risk of thromboembolic complications between patients with or without a history of cannabis use after total knee or hip arthroplasty. CI, confidence interval; M–H, Mantel–Haenszel; Random, random-effects model.

Meta-analysis of three studies (7, 9, 10) involving 96,971 individuals associated history of cannabis use with significantly higher incidence of venous thromboembolism (RR 1.48, 95% CI 1.34–1.63; I^2^ = 0%; Figure 4), and the evidence level was low (Appendix 1). Meta-analysis of four studies (7–10) involving 2,935,712 individuals linked cannabis use to significantly higher incidence of deep vein thrombosis (RR 1.42, 95% CI 1.19–1.70; I^2^ = 44%), for which the evidence level was low (Appendix 1); but it suggested no difference in incidence of postoperative pulmonary embolism between patients (7–10) with or without history of cannabis use (RR 1.14, 95% CI 0.77–1.68; I^2^ = 67%), though the evidence level was very low (Appendix 1).

### Postoperative pain and morphine consumption

**Figure 5:**
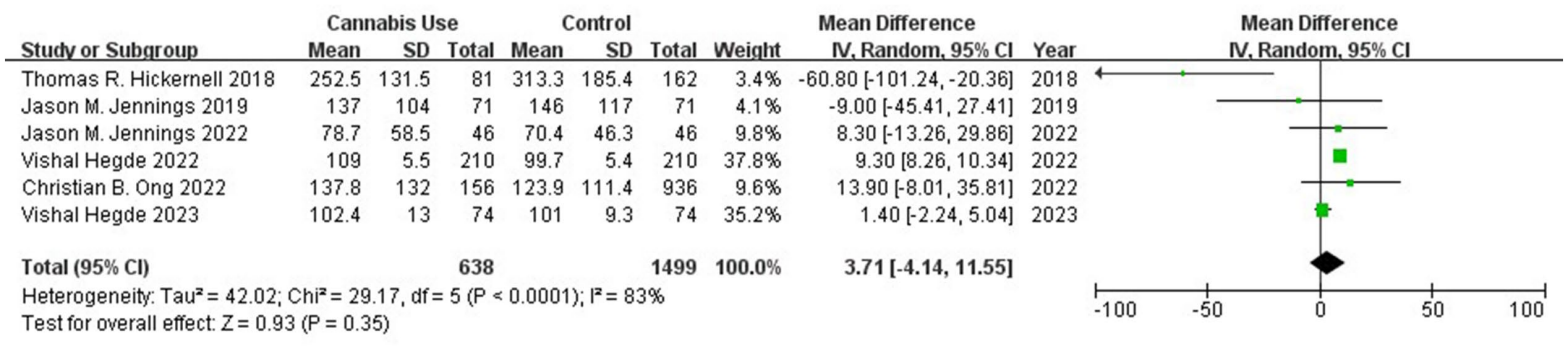
Forest plot of the mean difference of morphine consumption between patients with or without a history of cannabis use after total knee or hip arthroplasty. CI, confidence interval; Random, random-effects model.

Meta-analysis of six studies (11–16) involving 2,137 patients did not detect a significant difference in postoperative morphine consumption between patients with or without history of cannabis use (WMD 3.71, 95% CI-4.14 to 11.55; I^2^ = 83%; Figure 5), although the evidence level was very low (Appendix 1).

We were unable to meta-analyze differences in self-reported postoperative pain scores across the five cohort studies (11, 13, 22, 24, 27) that reported such data, because the studies assessed pain at different time points. Three retrospective studies (11, 22, 27) and one prospective study (13), which together involved 727 participants, reported no significant difference in postoperative pain between patients with or without history of cannabis use, while one retrospective cohort study (24) involving 149 participants linked cannabis use to significantly higher pain.

### Transfusion

**Figure 6:**
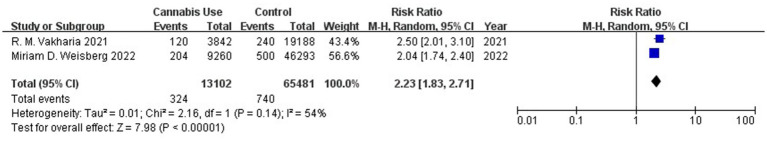
Forest plot of the risk of postoperative transfusion between patients with or without a history of cannabis use after total knee or hip arthroplasty. CI, confidence interval; M–H, Mantel–Haenszel; Random, random-effects model.

Meta-analysis of two studies (7, 9) involving 78,583 individuals linked history of cannabis use to significantly higher rate of postoperative transfusion (RR 2.23, 95% CI 1.83–2.71; I^2^ = 54%; Figure 6), for which the evidence level was moderate (Appendix 1).

### Hospitalization costs

Five retrospective cohort studies (7–10, 25) involving 2,936,780 individuals reported hospitalization costs related to joint replacement, but we were unable to meta-analyze the comparison between patients with or without history of cannabis use because the studies assessed different types of costs and sometimes differed in how they defined the same cost. All five studies reported that history of cannabis use was associated with higher hospitalization costs, which ranged from 16,938 to 59,570 USD for cannabis users and from 16,023 to 53,316 USD for controls.

## Discussion

This systematic review and meta-analysis appears to be the first attempt to synthesize available evidence about whether and how history of cannabis use affects complications, pain control, and other aspects following THA/TKA. Our analyses suggest that cannabis use is associated with greater risk of a range of negative outcomes, implying the need for special preoperative preparation and postoperative management of such patients.

The tetrahydrocannabinol in cannabis can cause feelings of euphoria that lead to addiction (28), it exerts analgesic and sedative effects by activating cannabinoid receptors 1 and 2, and it exerts adverse cardiovascular effects by activating the endocannabinoid system in the central nervous and cardiovascular systems (29). Phytocannabinoids can exacerbate infections by inhibiting the proliferation of T cells in peripheral blood (30, 31). These diverse effects of phytocannabinoids may help explain the plant’s observed negative effects on patient recovery after not only total joint replacement but other types of surgery as well (5). The effects of phytocannabinoids on the central nervous system may help explain why cannabis use increases risk of prosthetic complications: the plant compounds may decrease proprioception and impair motor coordination (32), which may contribute to risk of revision, mechanical loosening, periprosthetic fractures, and joint dislocation. Phytocannabinoids may reduce bone density by affecting mesenchymal stem cells (33, 34), which may also contribute to the observed increase in fracture risk. The observed higher risk of infection with cannabis use in our meta-analysis may reflect the ability of phytocannabinoids and other compounds in canabis smoke to suppress immune responses, analogously to compounds in cigarette smoke (35–38).

Consistent with a study linking history of cannabis use to greater risk of thrombotic complications in trauma patients (39), our meta-analysis confirmed an association between cannabis use and higher risk of venous thromboembolism and deep vein thrombosis. This association can be attributed to the ability of phytocannabinoids, acting through cannabinoid receptor 1, to stimulate the sympathetic nervous system while inhibiting the parasympathetic nervous system. These simultaneous processes increase heart rate and myocardial oxygen demand, leading to endothelial dysfunction and oxidative stress, which in turn may increase risk of myocardial infarction and stroke (40, 41). In addition, tetrahydrocannabinol may increase risk of thrombotic complications by activating platelets (42). Nevertheless, the evidence level for the effects of cannabis use on venous thromboembolism and deep vein thrombosis is not strong, highlighting the need for further research into these complications. Such research should verify our finding of no association between cannabis use and risk of pulmonary embolism, and it should explore why cannabis use appears to increase risk of thrombotic complications in veins but not arteries (39).

Nearly all studies in our review found no significant difference in postoperative pain between patients with or without history of cannabis use, which may help explain why our meta-analysis indicated no significant difference in postoperative morphine consumption between the two groups. This pooled result contrasts with previous work suggesting that cannabis use can reduce postoperative morphine consumption (43, 44). Nevertheless, the evidence level for this pooled result was very low, highlighting the need for rigorous research into whether cannabis use can mitigate pain after arthroplasty or other surgical procedures.

Our finding of an association between history of cannabis use and significantly higher rate of postoperative transfusion may reflect the higher risk of certain complications, such as bleeding. The observed association of cannabis use with higher hospitalization costs likely reflects the higher risk of numerous complications requiring prolonged treatment and management in hospital.

Our results should be interpreted with caution in light of several limitations. One is the fact that nearly all studies in our review were retrospective, and another is heterogeneity in how studies defined “history of cannabis use,” which probably contributed to the heterogeneity in several meta-analyses and weakened the evidence level. Indeed, none of the included studies reported sufficient detail about duration and intensity of cannabis use to allow us to evaluate dose–response relationships or control for confounding factors.

Despite these limitations, our systematic review of a large number of patients provides strong evidence that a history of cannabis use significantly increases risk of prosthetic, systemic and thromboembolic complications after THA/TKA, which in turn increases risk of transfusion and makes hospitalization more expensive. Whether cannabis us helps reduce pain and the need for morphine after joint replacement remains unclear. Future research should verify and extend our findings, particularly those with a lower evidence level. Clinicians may wish to use our findings to optimize preoperative preparation and postoperative management for patients with a history of cannabis use.

## Author contributions

GY: Conceptualization, Methodology, Writing – original draft. FL: Formal analysis, Investigation, Writing – review & editing. QW: Project administration, Data curation, Writing – review & editing. YL: Formal analysis, Funding acquisition, Writing – review & editing. JG: Funding acquisition, Project administration, Writing – review & editing. CY: Conceptualization, Writing – review & editing.

## Footnotes

^1^ www.zotero.org
