# Supplementary figures and images for: Association between history of cannabis use and outcomes after total hip or knee arthroplasty: a systematic review and meta-analysis
Source: Front Public Health. 2024 May 17;12:1377688. doi: 10.3389/fpubh.2024.1377688 (PMC11140086; doi:10.3389/fpubh.2024.1377688)

Appendix 1. Overall quality of evidence for each outcome from studies included.


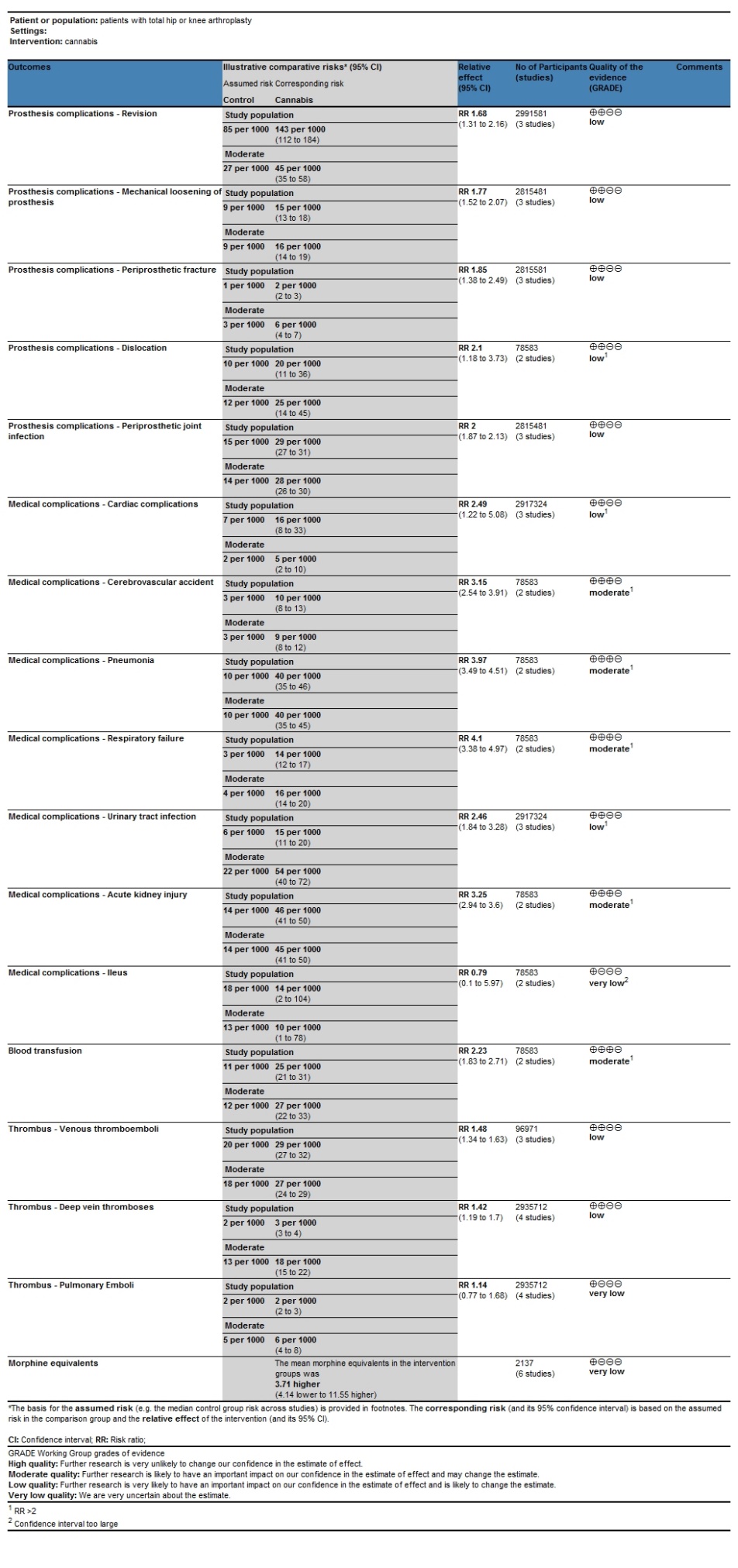

Supplement: Supplementary file 1 [file Table_1.docx]
